# Ultrasonic helical coil electrochemical reactor for simultaneous electrolysis–sonification–electrochemical polymerization, and applications for pollen cleaning

**DOI:** 10.1080/15685551.2021.2003557

**Published:** 2021-11-17

**Authors:** Kyoka Komaba, Hiromasa Goto

**Affiliations:** Department of Material Science, Faculty of Pure and Applied Sciences, University of Tsukuba, Tsukuba, Ibaraki, Japan

**Keywords:** Conductive polymers, electrochemical polymerization, microbubble, sonic electrochemical polymerization

## Abstract

Electrochemical polymerization of aniline by a combination of ultrasonic waves and electrolysis of water was performed. This method involves three processes: 1) creation of O_2_ micro bubbles produced by electrolysis of water on the anode side, 2) depolarization of the bubbles at the electrode surface via mechanical vibration using ultrasonic waves to diffuse ions in the electrolyte solution, and 3) progression of direct current (DC) electrochemical polymerization to yield a conductive polymer with fine pores on the surface. The diameter of the pores is on the micrometer scale and is similar in size to pollens. The combination of the electronic function of the conductive polymer and porous polymer surface can be applied as a method to collect allergens such as dust and flower pollens. Electrical adsorption and desorption of pollen was conducted with the porous polyaniline synthesized using a micro-bubble sonic-electrochemical preparation.

## 1. Introduction

Polyaniline (PANI) is one of the most promising conductive polymers because it can be prepared in water medium without inserting gas during the preparation process. PANI can be synthesized by oxidative chemical polymerization with oxidizing agents such as ammonium persulfate or H_2_O_2_ in the presence of an ion catalyst and electrochemical polymerization in water medium. Chemical polymerization can produce PANI in bulk or as a powder [1,2], whereas electrochemical polymerization allows for the production of an electroactive thin film deposited onto an electrode directly [3]. Thin PANI films on electrodes are useful for applications, such as battery [4], gas sensor [5], and anti-corrosion films [6].

Sonoelectrochemistry is the use of ultrasonic waves when conducting an electrochemical process. The combination of ultrasonic waves and electrochemistry can enhance the efficiency of a reaction. Ultrasonic waves provide an increase in temperature and diffusion of solution by cavitation [7]. Cavitation can adjust diffusion in solution and the morphology of products in chemical reactions [8]. Furthermore, sonoelectrochemistry offers many advantages for chemical reactions [9], reduction of electrode fouling [10], and improvement of current efficiency [11]. Pollet *et al*. succeeded in developing a novel method for the preparation of polymer electrolyte membrane fuel cell (PEMFC) electrodes using sonoelectrochemistry for the first time. PEMFC electrodes prepared by sonoelectrochemical methods perform better in galvanostatic experiments compared to those prepared with silent conditions and conventional methods [12,13]. Ganesan *et al*. synthesized polyaniline nanoparticles (20–40 nm) by a sonoelectrochemical method. Ultrasonic waves were used for irradiation during the electrochemical polymerization of aniline as a monomer to increase the polymerization efficiency. The electrochemical method can control the size of the resultant polymer particle by regulating the applied potential or current density. Moreover, ultrasonic waves can increase the efficiency of reactions through the generation of high-temperature bubbles by cavitation [14]. Electrochemical synthesis of microporous polyaniline films using a combination of electrochemical polymerization, sonification, and foam template to obtain polyaniline having fine microporous structure was developed [14].

We developed micro-bubble sonic-electrochemical preparation with sandwiched two-electrode, and application of ultrasonic from the outside of the reaction vessel without use of soaps prior to the present research. In the present study, we assembled a new type electrochemical polymerization reactor consisting of a helical electrode and ultrasonic generator in the center of the reactor for micro-bubble sonic-electrochemical polymerization to prepare a porous polymer. Generation of air bubble during the electrochemical polymerization is carried out by electrolysis of water. Porous structures can be prepared to mimic the shape of micro bubbles. Pore size can be controlled by the amount and size of micro bubbles during the polymerization process by tuning the applied voltage. Various methods to produce micro bubbles have been studied. Micro bubbles can be generated by chemical reactions, injection of air at high pressure, and cavitation. Although electrolysis of water is convenient as a method to produce micro bubbles, this electrolysis can only occur with the application of high voltage to electrodes in water medium in the presence of a supporting salt. Micro bubbles in water at the anode side comprise O_2_ gas. When electrochemical polymerization and electrolysis occur simultaneously, polymer is deposited on the anode side with a discharge of O_2_ bubbles to produce a conductive porous polymer. However, O_2_ bubbles tend to remain at the anode side and suppress electrodeposition of polymer. To solve this problem, we used the acoustic vibration of ultrasonic waves to release and diffuse O_2_ bubbles from the electrode surface during polymerization. This method also allows for the sequential progress of electrochemical polymerization in a fresh O_2_ environment. Aniline was used as the monomer in the present research.

The porous PANI synthesized with this method can be utilized in electrical cleaning systems that trap micro particles. Pollen spheres are on the micrometer scale with a cationic charge. Therefore, pollens can be captured by porous polymers with an anionic charge through mechanical and electrostatic functions. Moreover, reversing the current direction can desorb pollens out of the polymer surface.

## 2. Experiment

### 2.1. Materials

Aniline and iodine were purchased from Fujifilm Wako Pure Chemical Industries (Tokyo). Sulfuric acid was obtained from Nacalai Tesque (Kyoto). *Cryptomeria japonica* (Japanese cedar, pollen) was obtained from the Institute of Tokyo Environmental Allergy (Tokyo). All materials were used as received.

### 2.2. Techniques

Ultrasonic waves were generated by a portable ultrasonic cleaner CE-9600 (Blumway, Shenzhen, China). Optical texture observations were conducted using a high-resolution polarizing microscope (ECLIPS LV 100, Nikon) with an LU Plan Fluor lens and a CFIUW lens (Nikon, Tokyo) with circular polarized differential interference contrast microscopy (C-DIM) unit. Scanning electron microscopy (SEM) observations were conducted with a JSM-7000 F instrument (JEOL, Akishima, Japan). Fourier transform infrared spectra were obtained using an FT/IR-4600 spectrometer (JASCO, Tokyo, Japan) by the KBr method. Reflection spectroscopy measurements were performed on a V670 with an ARMN-735 (JASCO) instrument. Cyclic voltammetry (CV) measurements were obtained by repeated potential cycling using an ECO CHEMIE μAUTOLAB TYPE III. Electron spin resonance (ESR) measurements of the solid sample, which was packed in a 5-mm quartz tube, were performed using a JEOL JES TE-200 spectrometer in X-band (Tokyo, Japan).

### 2.3. Preparation of PANI_ULTRA_

**Figure 1.**
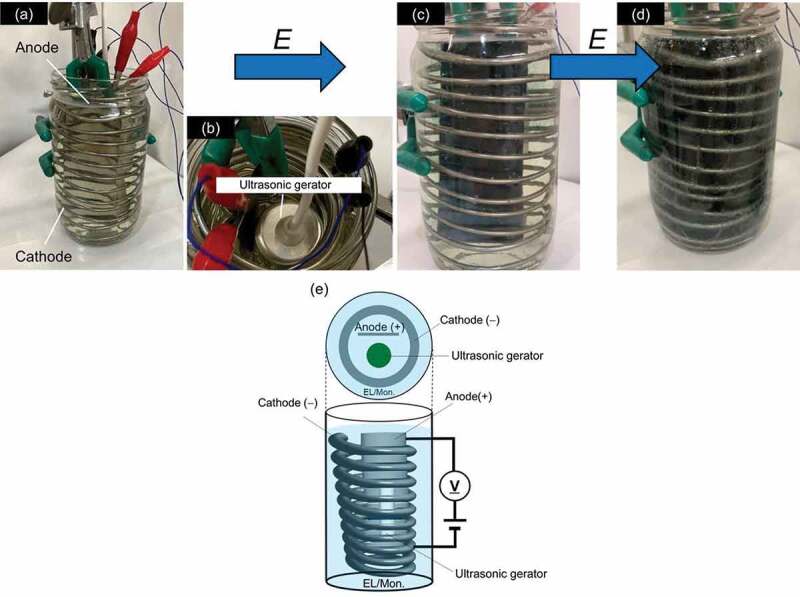
An ultrasonic helical coil electrochemical reactor for micro-bubble sonic-electrochemical polymerization: (a) Setup of the instrument for micro-bubble sonic-electrochemical polymerization, (b) Top view, (c) Polymerization for 3 min, (d) 4 min. (e) Overview of electro-circuit. EL/Mon.: Electrolyte containing monomer

**Scheme 1.**
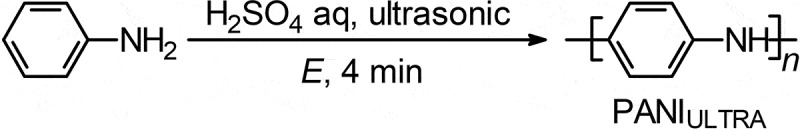
Preparation of polyaniline by micro-bubble sonic-electrochemical polymerization

Polyaniline was prepared by micro-bubble sonic-electrochemical polymerization. An electric field of 5–6 V (DC) was applied across two stainless-steel electrodes in the presence of aniline and sulfuric acid. The electrolyte was prepared by dissolving aniline (5.72 g) and sulfuric acid (5.30 g) in water (500 mL). Application of excess voltage causes electrolysis of water. In general, bubbles produced by the electrolysis of water cover electrodes, resulting in electrolytic polarization of the electrodes. The polarization depressed further progress of the electrochemical reaction. Application of the ultrasonic allows surface of the electrode to avoid this polarization. (Scheme 1, Figure 1). Electrochemical polymerization was conducted for 4 minutes at room temperature to be applied voltage (5 V) under ultrasonic waves. The polymer thus prepared was abbreviated as PANI_ULTRA_. The frequency of ultrasonic wave was ca. 50 kHz.

This polymerization was conducted with a custom-made electrochemical polymerization device, as shown in Figure 1. The polymerization vessel contained an anode and a helical cathode coil in an aqueous aniline/sulfuric acid solution. The electrodes were made of stainless steel (Figure 1(a)). An ultrasonic generator was located at the center of the vessel (Figure 1(b), top view). After application of voltage, O_2_ and H_2_ gas bubbles were generated by the electrolysis of water, and electrochemical polymerization-deposition occurred on the cylinder anode. Aniline was gradually polymerized on the electrode. The color gradually changed to green as growth of the polyaniline films on the electrode. After 3 minutes, the entire electrode changed to dark green. Polymerization was conducted for a total of 4 min.

## 3. Results and discussion

### 3.1. Surface structure

**Figure 2.**
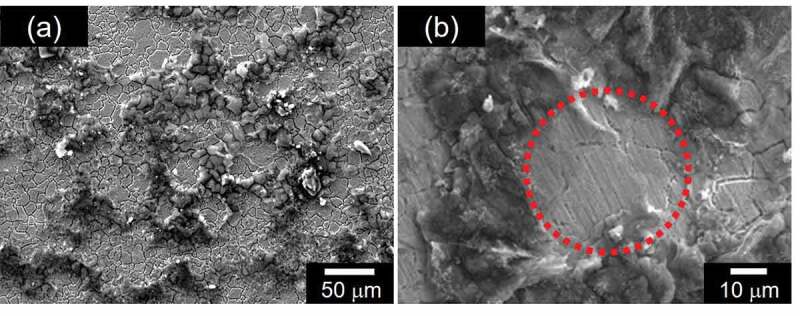
SEM images of PANI_ULTRA_. (a) Low magnification (×300). (b) High magnification (×1000))

Figure 2 shows the image of SEM for PANI_ULTRA_ as prepared, with no coating such as Au. The porous structure appeared on the PANI_ULTRA_ film (Figure 2(a)). The holes are randomly placed in the PANI_ULTRA_ film. Some have multiple holes overlapping. A small circle on the surface is shown in Figure 2(b). The diameter of the pore was ca. 40 µm, which is comparable to that of the pollen.

### 3.2. Fourier transform infrared spectroscopy and reflection spectroscopy

**Figure 3.**
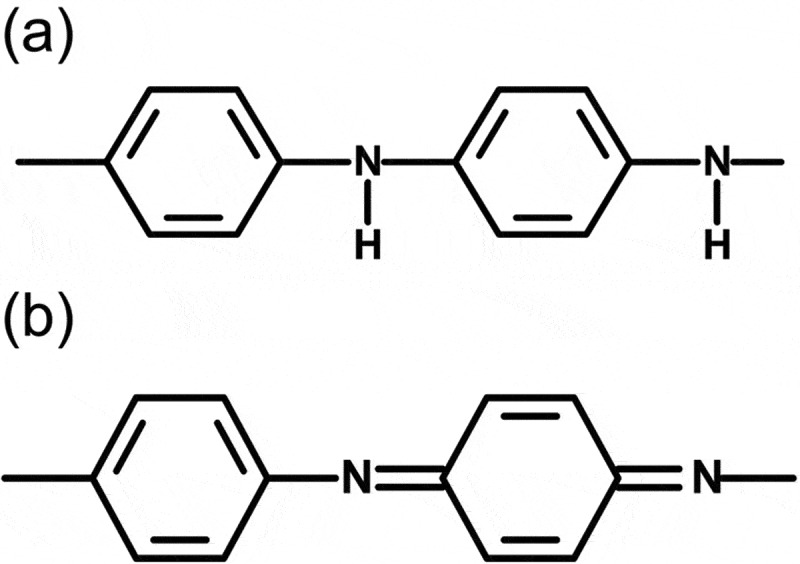
Benzenoid and quinonoid units of polyaniline (PANI): (a) benzenoid structure and (b) quinonoid structure

**Figure 4.**
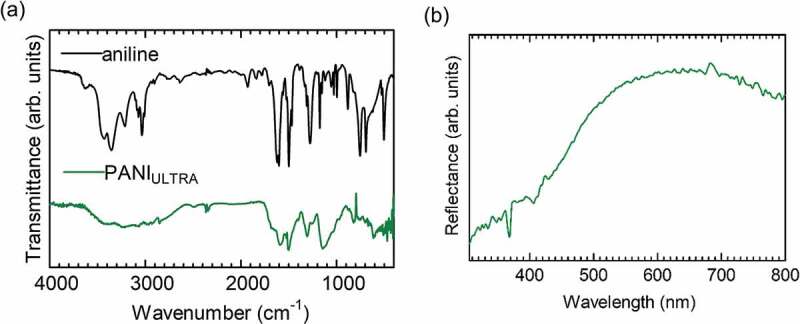
(a) IR spectra. (b) Reflectance spectrum of PANI_ULTRA._

PANI prepared by the electrochemical method shows emeraldine salt (ES, doped form). Tang et al. reported the IR absorption of pure PANI in detail, and we considered the chemical structure in accordance with their results [15]. The ES has both benzenoid (B) and quinonoid (Q) structure in the main chain, as shown in Figure 3. The entire main chain of PANI comprises a combination of quinonoid and benzenoid sequences. Fourier transform infrared (FT-IR) spectroscopy measurements for PANI_ULTRA_ were conducted with the KBr method, and the results are displayed in Figure 4(a). Assignments of the transmittance signals are summarized in Table 1. Aniline shows the several signals derived from NH stretching around 3300 cm^−1^. Meanwhile, a signal for NH stretching of the aniline unit in the benzenoid structure was observed only at approximately 3300 cm^–1^. Furthermore, stretching vibrations of N=Q=N, N–B–N, QBQ and BBB were shown at 1589, 1499, 1307 and 1144 cm^−1^ for PANI_ULTRA_ only. These are derived from vibrations of the main chain skeleton. IR measurements confirm that the chemical structure of the resultant polymer is in accordance with the fundamental structure of polyanilines prepared by the general method.

**Table 1.** IR absorption signals^a^ of PANI_ULTRA._

	Wavenumber (cm^−1^)						
	ν_NH_	ν_CH_	δ_NH_	ν_N=Q=N_	ν_N-B-N_	ν_QBQ_	ν_BBB_
aniline	3428 3352	3036	1620				
PANI_ULTRA_	3171	—		1589	1499	1307	1144
^a^ν = stretching vibration, δ = bending vibration.							

Reflection spectrum for PANI_ULTRA_ was examined. The spectrum shape was shown in Figure 4(b). A broad reflection was shown at ca. 600 nm as green color range reflection, confirming PANI_ULTRA_ has green color.

### 3.3. Cyclic voltammetry (CV)

**Figure 5.**
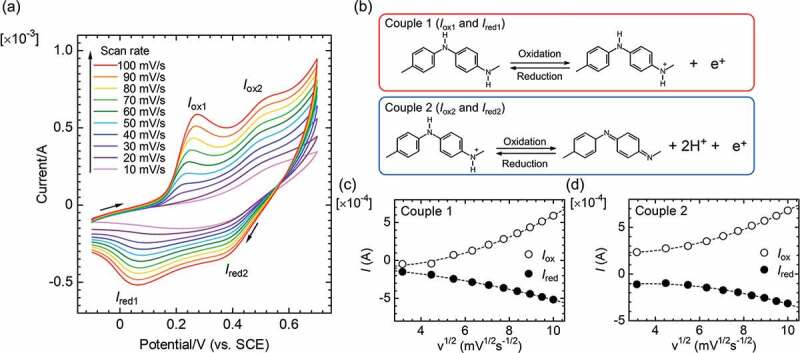
Cyclic voltammetry (CV) result for PANI_ULTRA_ in 0.1 M H_2_SO_4_aq vs. saturated calomel electrode (SCE) reference electrode. (a) Entire CV curve. (b) Change in molecular structure in the redox. (c) Signal plots as a function of the square root of the scan rate for Couple 1. (d) Signal plots as a function of the square root of the scan rate for Couple 2

CV measurements were obtained for PANI_ULTRA_ deposited on the stainless electrode in 0.1 M H_2_SO_4_aq. A Pt electrode was used as a counter electrode, and a saturated calomel electrode (SCE) electrode was used as a reference electrode. Figure 5 displays the CV results with a change in scan rate from 10 to 100 mV/s. The increase in scan rate increased the current density for PANI_ULTRA_ due to increased diffusion of ions in the electrolyte solution. The CV curve shows two oxidation signals and two reduction signals in the redox cycle. The first set of a redox couple (Couple 1; *I*_ox1_ and *I*_red1_) is associated with a change between leucoemeraldine base and oxidized emeraldine. The second set of redox current signals (Couple 2; *I*_ox2_ and *I*_red2_) is associated with a change between emeraldine and pernigraniline form [16].

Plots of the oxidation peak current (*I*_ox1_, *I*_ox2_) and the reduction peak current (*I*_red1_, *I*_red2_) as a function of the square root of the scan rate for Couples 1 and 2 are shown in Figures 5(c-d), respectively. The plots of oxidation signals (*I*_ox1_, *I*_ox2_) and reduction signals (*I*_red1_, *I*_red2_) indicate that the signal currents increase with scan rate (v: velocity).

### 3.4. Electron spin resonance (ESR)

**Figure 6.**
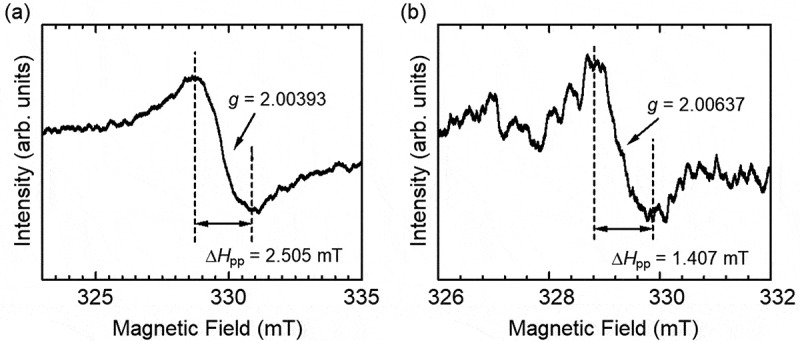
Electron spin resonance (ESR) spectra of (a) PANI_ULTRA_ and (b) iodine-doped pollen

Figure 6 shows the results of ESR. PANI_ULTRA_ showed the signal derived from radicals in the conductive polymer. The g value was 2.00393, indicating that nitrogen radicals due to charge carrier polarons (radical cations) were present. Vapor-phase iodine-doped pollen was also measured for ESR. Pollen is known to induce allergic reactions when combined with car exhaust comprising NO_x_ and SO_x_, and these materials attract electrons. *Cryptomeria japonica* (Japanese cedar, pollen) was doped with iodine. This doped pollen contained oxygen radicals (g value = 2.00637). Oxygen radicals may capture pollen electrically by injecting electrons into the electrode. The cationic iodine-doped pollen can be adsorbed on the PANI_ULTRA_ in the emeraldine base (EB) state as in a half-doped form.

### 3.5. Electrical cleaning system

#### 3.5.1. Pollen absorption in water medium

**Figure 7.**
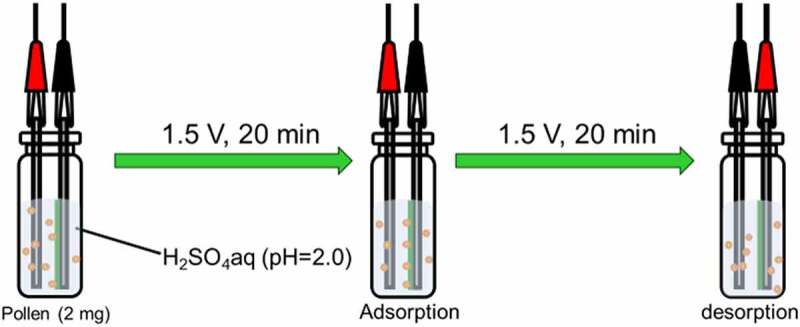
Electric adsorption of pollen particle onto PANI_ULTRA_ with voltages in water medium

Electrical adsorption and desorption of pollen in water medium were conducted (Figure 7). Pollen (2 mg) was added to aqueous sulfuric acid (pH = 2). Porous polyaniline deposited on the stainless-steel electrode was set into a vial filled with H_2_SO_4_ aq. A stainless-steel electrode with no polyaniline was set into the vial filled with H_2_SO_4_ aq. The distance between the electrodes was ca. 5 mm to face each other. First, a DC voltage of 1.5 V for 20 min was applied to capture pollen on the cathode side. Next, a voltage of 1.5 V for 20 min was applied to desorb pollen captured in the first step.

We would like to change Figure 7. Please find revised Figure 7 and replace it.

**Figure 8.**
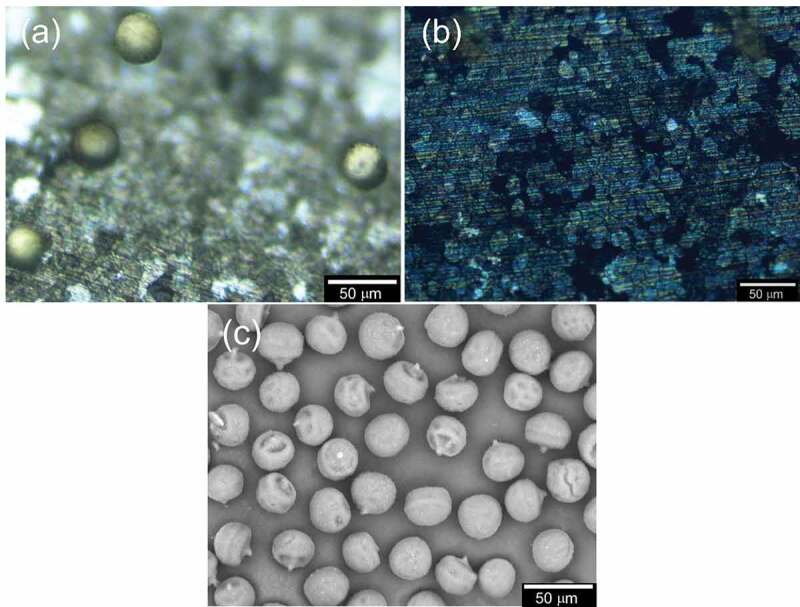
C-DIM images of PANI_ULTRA_: (a) Electrical adsorption step and (b) Electrical desorption. (c) SEM image of pollen used in this study

The electrode was observed by C-DIM in each step (Figure 8). Figure 8(a) shows electrical absorption of pollens. Figure 8(b) displays the pollen desorption after reversing the application of voltage. Figure 8(c) shows the SEM image of pollen used in this study. The diameter is ca. 25 µm.

#### 3.5.2. Pollen absorption in atmosphere

Electric adsorption of pollen particles on PANI_ULTRA_ in the air was also conducted. Previous study suggested that diesel exhaust particles from vehicle and pollen affect allergic [17,18]. The pollen having free radical affects some allergic symptoms for people. The polyaniline modified electrode having pollen capture function may contribute to purification of air.

**Figure 9.**
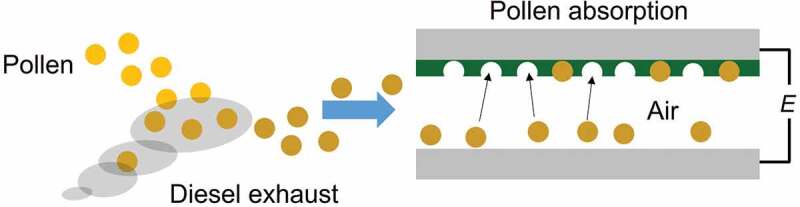
Schematic diagram of electrical adsorption of pollen particles PANI_ULTRA_ with high voltage in the air

Porous polyaniline deposited on the stainless-steel electrode was set polyaniline on top and stainless-steel electrode with no polyaniline was set to the face each other. The distance between the electrodes was ca. 5 mm. The outside was covered with transparent acrylic case. Pollen was set on the lower electrode. Then, a high AC voltage was applied (~20 kV) for about 20 seconds, and then the stainless-steel electrode with porous polyaniline was taken out (Figure 9).

**Figure 10.**
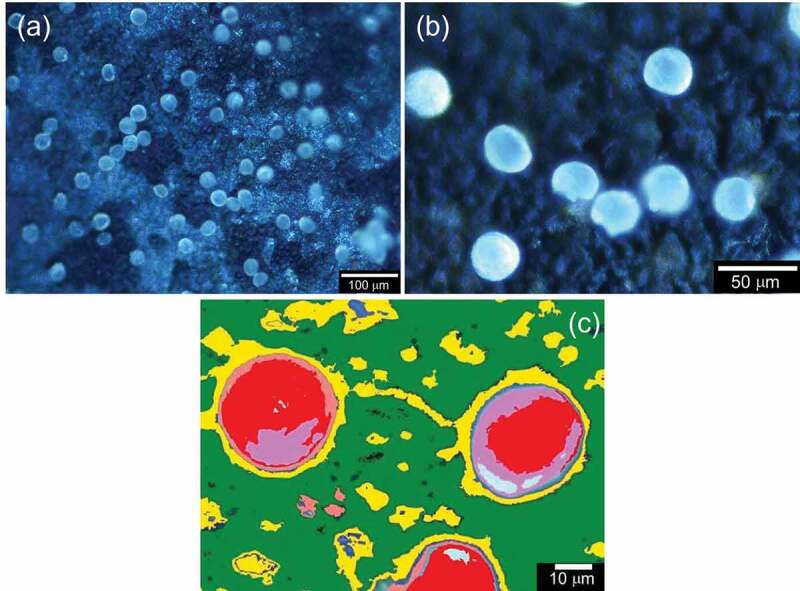
C-DIM image of PANI_ULTRA_: (a) electrical adsorption in the air (low magnification) and (b) electrical desorption (high magnification). (c) Computer graphic image processing to enhance the adsorption from a C-DIM image of the surface of PANI_ULTRA_ with pollen particles

The electrode was observed by the C-DIM. Figure 10(a,b) shows the C-DIM image of PANI_ULTRA_ with pollens on the surface, which is deposited from the air by a high voltage electric field. PANI_ULTRA_ in the emeraldine base (EB) state as in a half-doped form captured cationic iodine-doped pollen. Figure 10(c) displays a computer graphic image processing to enhance the C-DIM image of the surface of PANI_ULTRA_ with adsorbed pollen particles. The yellow and red areas in Figure 10(c) are holes and pollens, respectively, indicating that the pollens are captured by the holes on the porous surface.

## 4. Conclusion

Porous PANI was prepared by micro-bubble sonic-electrochemical polymerization. The process of micro-bubble sonic-electrochemical polymerization involves 1) generation of the O_2_ micro-bubble structure, 2) diffusion of bubbles on the surface of the electrode by depolarizing with the vibration of ultrasonic waves, and 3) electrochemical polymerization and deposition onto the electrode to form an electroactive polymer film with micrometer-sized pores on the surface. Thus, the PANI electrode was obtained with pores on the micrometer scale.

Porous PANI electrodes show reversible redox activity. Electrical adsorption and desorption of pollens were successful. Therefore, cleaning systems utilizing this method may capture not only pollens but also pollens with oxygen radicals.

## References

[1] Mohammad-Rezaei R, Massoumi B, Abbasian M, et al. Electrically conductive adhesive based on novolac-grafted polyaniline: synthesis and characterization. J Mater Sci Mater Electron. 2019;30(3):2821–2828.

[2] Abel SB, Yslas EI, Rivarola CR, et al. Synthesis of polyaniline (PANI) and functionalized polyaniline (F-PANI) nanoparticles with controlled size by solvent displacement method. Application in fluorescence detection and bacteria killing by photothermal effect. Nanotechnology. 2018;29:125604. 29355838 10.1088/1361-6528/aaa99a

[3] Liu S, Liu L, Guo H, et al. Electrochemical polymerization of polyaniline-reduced graphene oxide composite coating on 5083 Al alloy: role of reduced graphene oxide. Electrochem Commun. 2019;98:110–114.

[4] Ma X, Zou S, Tang A, et al. Three-dimensional hierarchical walnut kernel shape conducting polymer as water soluble binder for lithium-ion battery. Electrochim Acta. 2018;269:571–579.

[5] Matindoust S, Farzi A, Baghaei Nejad M, et al. Ammonia gas sensor based on flexible polyaniline films for rapid detection of spoilage in protein-rich foods. J Mater Sci Mater Electron. 2017;28(11):7760–7768.

[6] Wu J-W, Wang T-L, Lin W-C, et al. Anti-Corrosion Characteristics of Electrodeposited Self-Doped Polyaniline Films on Mild Steel in Low Acidity. Coatings. 2018;8(5):155.

[7] Pollet BG, Ashokkumar M. Short Introduction to Sonoelectrochemistry. Pollet BG, Ashokkumar M,editors. Introd Ultrasound Sonochemistry Sonoelectrochemistry [Internet]. Cham:Springer International Publishing.2019 cited 2019 Dec 30 21–39.Available from 10.1007/978-3-030-25862-7_2.

[8] Hujjatul Islam M, Paul MTY, Burheim OS, et al. Recent developments in the sonoelectrochemical synthesis of nanomaterials. Ultrason Sonochem. 2019;59:104711. 31421622 10.1016/j.ultsonch.2019.104711

[9] Islam MH, Burheim OS, Pollet BG. Sonochemical and sonoelectrochemical production of hydrogen. Ultrason Sonochem. 2019;51:533–555. 30442455 10.1016/j.ultsonch.2018.08.024

[10] Garbellini GS, Salazar-Banda GR, Avaca LA. Sonovoltammetric determination of 4-nitrophenol on diamond electrodes. J Braz Chem Soc. 2007;18(6):1095–1099.

[11] Ruecroft G, Hipkiss D, Ly T, et al. Sonocrystallization: the Use of Ultrasound for Improved Industrial Crystallization. Org Process Res Dev. 2005;9(6):923–932.

[12] Pollet BG. A novel method for preparing PEMFC electrodes by the ultrasonic and sonoelectrochemical techniques. Electrochem Commun. 2009;11(7):1445–1448.

[13] Pollet BG. The Use of Power Ultrasound for the Production of PEMFC and PEMWE Catalysts and Low-Pt Loading and High-Performing Electrodes. Catalysts. 2019;9(3):246.

[14] Ganesan R, Shanmugam S, Gedanken A. Pulsed sonoelectrochemical synthesis of polyaniline nanoparticles and their capacitance properties. Synth Met. 2008;158(21–24):848–853.

[15] Tang J, Jing X, Wang B, et al. Infrared spectra of soluble polyaniline. Synth Met. 1988;24(3):231–238.

[16] Song E, Choi J-W. Conducting Polyaniline Nanowire and Its Applications in Chemiresistive Sensing. Nanomaterials. 2013;3(3):498–523. 28348347 10.3390/nano3030498 PMC5304646

[17] Chehregani A, Kouhkan F. Diesel exhaust particles and allergenicity of pollen grains of Lilium martagon. Ecotox Enviro Safe. 2008;69(3):568–573 10.1016/j.ecoenv.2007.05.007 17597207

[18] Muranaka M. Investigation of clinical epidemiological study supporting the link between increased cedar pollen allergy and diesel exhaust particulates (Japanese). Prep Jpn Soc Atmos Env. 2000;41:471.

